# Reflecting a crisis reaction: Narratives from patients with oesophageal cancer about the first 6 months after diagnosis and surgery

**DOI:** 10.1002/nop2.348

**Published:** 2019-08-02

**Authors:** Ylva Hellstadius, Marlene Malmström, Pernilla Lagergren, Magnus Sundbom, Anna Wikman

**Affiliations:** ^1^ Regional Cancer Centre Stockholm Sweden; ^2^ Department of Clinical Sciences Skåne University Hospital, Lund University Lund Sweden; ^3^ Department of Molecular Medicine and Surgery Karolinska Institute Stockholm Sweden; ^4^ Department of Surgical Sciences Uppsala University Uppsala Sweden; ^5^ Department of Women's and Children's Health Uppsala University Uppsala Sweden

**Keywords:** adaptation, interviews, nurses, nursing, oesophageal cancer, qualitative

## Abstract

**Aim:**

The aim of the study was to describe patients' experiences of emotional adaption following treatment for oesophageal cancer from diagnosis to 6 months after surgery.

**Design:**

A qualitative interview study using an inductive approach was carried out.

**Methods:**

Participants were recruited from two university hospitals in Sweden. Ten patients who had been operated for oesophageal cancer with curative intent 6 months earlier and consented to participate in the study were included. Patients who had a disease recurrence were not eligible for inclusion. Participants were interviewed with a semi‐structured interview approach. Data were analysed using qualitative content analysis.

**Results:**

One overarching theme was identified; *Experiencing a crisis reaction*, which comprised three key categories; (a) *From emotionally numb to feeling quite alright*; (b) *From a focus on cure to reflections about a whole new life*; and (c) *From a severe treatment to suffering an emaciated, non‐compliant body*, derived from 14 distinct sub‐categories.

**Conclusion:**

This study highlights the process of emotional adaptation following oesophageal cancer surgery that patients describe when reflecting back on the first 6 months postoperatively pointing to a crisis reaction in this early postoperative period.

## 1 INTRODUCTION

Oesophageal cancer is the sixth most common cause of cancer deaths worldwide. Around 600 patients are diagnosed in Sweden yearly and about 500,000 new cases are diagnosed worldwide (Torre et al., 2015). There are two histological types, squamous cell carcinoma and adenocarcinoma, with distinct risk factor profiles. For oesophageal squamous cell carcinoma, tobacco smoking and excess alcohol use are the main risk factors. Whereas for adenocarcinoma, gastro‐oesophageal reflux disease and obesity are main risk factors (Xie & Lagergren, 2018). Being diagnosed with oesophageal cancer has been described in interview studies as “coping with a death sentence” (McCorry, Dempster, Clarke, & Doyle, 2009). The vital function of the oesophagus, that is, transporting food from the mouth to the stomach, has a great physical and psychological impact on patients who suffer from a tumour in this area (Clarke, McCorry, & Dempster, 2011). Oesophageal cancer has an asymptomatic onset allowing tumours to grow quite large before being discovered (Bird‐Lieberman & Fitzgerald, 2009). Consequently, the overall prognosis is poor and only 30% of patients are eligible for curatively intended treatment at the time of diagnosis (Coupland et al., 2013). Further, for those patients who do experience symptoms, the most common symptom of oesophageal cancer is dysphagia, that is, swallowing difficulties (Daly et al., 2000). This symptom makes the pre‐operative time for patients problematic, due to poor swallowing function and weight loss and often in combination with the side effects of chemotherapy (Martin, Jia, Rouvelas, & Lagergren, 2008).

### 1.1 Background

To date, the curatively intended treatment for oesophageal cancer is surgery in combination with chemotherapy (Allum et al., 2011). The anatomic location of the oesophagus, embedded in the thorax, close to vital organs, makes the surgical procedure challenging and the risk of complications during the operation and postsurgery is high (Smith et al., 2014). Following the surgical procedure, a new challenge begins for the patients; adaptation to a new life situation with altered eating habits, due to the gastric tube replacing the resected oesophagus. In postoperative interview studies with patients who have undergone oesophagectomy for cancer, this has been described as “when moving on becomes a struggle” (Malmström, Ivarsson, Johansson, & Klefsgård, 2013). Despite treatment with curative intent, the long‐term prognosis remains poor and the risk of tumour recurrence is high during the first year (Lou et al., 2013). Further, the postoperative pathway is also challenging for this patient group due to poor eating conditions affecting their social life (Martin, Lagergren, Lindblad, Rouvelas, & Lagergren, 2007; Wainwright, Donovan, Kavadas, Cramer, & Blazeby, 2007). Good nutritional status in patients with cancer is not only a prerequisite for successful rehabilitation and long‐term health‐related quality of life (Climent et al., 2017), but also carries a symbolic meaning indicating “life” for many people (Derogar & Lagergren, 2012). Therefore, it is of utmost importance to investigate the psychological consequences of surgically treated patients with oesophageal cancer due to the fatal nature of the disease, the challenging treatment pathway, the potential for severe pre‐ and postoperative symptoms (e.g. swallowing difficulties and poor nutritional status) and the changed life situation requiring adaptation to long‐term symptoms (e.g. gastro‐oesophageal reflux, diarrhoea, dumping) (Derogar & Lagergren, 2012; Malmström et al., 2013; Schandl, Lagergren, Johar, & Lagergren, 2016). There is growing evidence that emotional distress among patients with cancer is not only associated with poor adjustment (Antoni, 2013), but also suicidal thoughts (Walker et al., 2008) and increased risk of cancer mortality (Russ et al., 2012). Moreover, research investigating trajectories of adjustment to cancer survivorship addresses that recovery is not one fixed model or a dichotomized experience of “distress” or “no distress”. Patient‐reported emotional aspects such as worry, depression and post‐traumatic growth have been shown to be associated with patients' narratives describing the recovery path (Ratcliff, Naik, Martin, & Moye, 2018).

Previous large‐scale quantitative studies have indicated that patients with oesophageal cancer suffer significant emotional distress from diagnosis up to 5 years postsurgery (Hellstadius et al., 2014), with an increased risk of severe psychological morbidity during the first 3 months postdiagnosis (Fang et al., 2012). Studies have also shown that anxiety symptoms are highest pre‐operatively, levelling off at 6 months, whereas depressive symptoms remain elevated from 6 months and onwards up to one‐year post‐treatment (Hellstadius et al., 2017). The early postoperative course has previously been described by patients with oesophageal cancer as a particularly demanding time period (Olsson, Bosaeus, & Bergbom, 2010) mainly due to the severe physical symptom burden caused by the altered anatomy of the gastrointestinal tract (Wainwright et al., 2007). At 6 months, most patients who have undergone surgery for oesophageal cancer have been discharged from inpatient specialist care and discharged back to their homes for recovery. Previous studies have shown that patients with oesophageal cancer report impaired health‐related quality of life (HRQOL) including aspects of emotional functioning at this time point (Djärv, Lagergren, Blazeby, & Lagergren, 2008), indicating that the first few months postoperatively might be a particularly vulnerable time during the course of recovery with increased needs for support. The aim of the present study was to describe patients' experiences of emotional adaption following treatment for oesophageal cancer from the time of diagnosis to 6 months after surgery.

## 2 METHOD

### 2.1 Design

This was a descriptive qualitative interview study using an inductive approach.

### 2.2 Participants and procedure

Participants were identified via the upper gastrointestinal clinics at two university hospitals in Sweden, where they had been operated for oesophageal cancer with curative intent approximately 6 months earlier. Patients diagnosed with oesophageal cancer and treated with curative intent (i.e. surgery only, or surgery and chemotherapy in combination) and who were able to describe their lived experience during the first 6 months following diagnosis were included in the study. Interviews were planned to be carried out as close to 6 months postoperatively as possible. Participants with cancer recurrence were not eligible for inclusion. After identification via the hospital clinics, a letter with full details about the study was sent to the home address of potential participants. Potential participants were offered the opportunity to opt‐out of being contacted and receiving further information about the study by completing and returning a pre‐paid reply slip (or by e‐mail or telephone call). Two weeks after the information letter had been sent out, participants who had not opted‐out were contacted via telephone by the first author (Y.H). During the phone call, the aim of the study was fully explained and further information was given. If a potential participant agreed to participate, a time and place for the interview was agreed between the participant and interviewer. Before the start of each interview, written informed consent was obtained. Twenty‐one potential participants (consecutively numbered 1–21) had undergone surgery for oesophageal cancer 6 months prior to the time of the interviews and were eligible for inclusion in the study (four women, 19%; 17 men, 81%). Of those, three declined participation, two were living too far away, four were not reachable by phone and one was too unwell to participate at the time of the interview. Thus, 10 (48%) participants were included in the study (participants with study numbers 04, 05, 06, 08, 12, 13, 16, 17, 19 and 20 were included).

### 2.3 Data collection

Data were collected through in‐depth, face‐to‐face interviews. The interviews were carried out in the participants' homes, by the first author (Y.H.) and were audio‐recorded. An interview guide was used to follow the same structure through all interviews. The interview time varied between 32–54 min (median time: 46 min). Open‐ended questions addressing participants experiences at the time of diagnosis, during and after the treatment, at present and the future were used to explore participants' experiences of emotional adaptation from the time of diagnosis until 6 months after surgery. In addition, probing questions, such as “can you tell me more?” or “can you explain in more detail?” were asked to increase the richness of the material (Appendix S1). The interviews were carried out until no new information related to the research question was addressed from the participant.

### 2.4 Analysis

All audio‐recorded interviews were transcribed verbatim. To attend both the manifest and latent aspects of the material, limiting data reduction to the aspects of relevance to the research question, data were analysed with an inductive framework using qualitative content analysis (Graneheim & Lundman, 2004). Conventional content analysis was used as the aim of the study was to describe a phenomenon, in the present study the patients' experiences of adaptation from the diagnosis of oesophageal cancer to 6 months after curatively intended treatment (Hsieh & Shannon, 2005). An inductive approach was used to reflect frequently reported patterns in the material and bring them together as a whole. The first and last authors (Y.H. & A.W.) conducted the analysis in parallel and then discussed the interpretations with all co‐authors. The responses were read repeatedly to gain an overall understanding. Meaning units were identified, defined as words and sentences containing aspects related to the research question through common content and context. Meaning units were condensed, that is, shortened without losing the content and context. Condensed meaning units were each assigned descriptive codes, functioning as labels of the content. The various codes were compared based on differences and similarities and sorted into 14 sub‐categories and three categories, which constitute the manifest content. A reflective process and discussions between all authors resulted in agreement about how to sort the codes. Finally, the underlying meaning, that is, the latent content of the categories was formulated into a theme. A theme, like a “red thread”, representing an underlying meaning through condensed meaning units, codes or categories, on an interpretative level is seen as an expression of the latent content of the text (Graneheim & Lundman, 2004). Representative quotations were chosen to illustrate the research findings (Sandelowski, 1991).

## 3 RESULTS

Participants' age and sex are shown in Table 1. Eight participants were men (80%), and the median age was 66.5 years (range 48–80) at the time of the interview. All participants had been treated with combination therapy, that is, neoadjuvant chemotherapy and surgery with curative intent 6 months previously (median = 6, range 5–7). None of the participants had any history of psychiatric morbidity (self‐reported) prior to the oesophageal cancer diagnosis. None had been prescribed psychotropic drugs or received any other psychological treatment during the postoperative treatment pathway (self‐reported).

**Table 1:** Age and sex of participants

Participant number	Age	Sex
#04	65	Male
#05	74	Male
#06	68	Male
#08	73	Male
#12	48	Male
#13	52	Female
#16	68	Male
#17	80	Male
#19	61	Female
#20	51	Male

**Figure 1:**
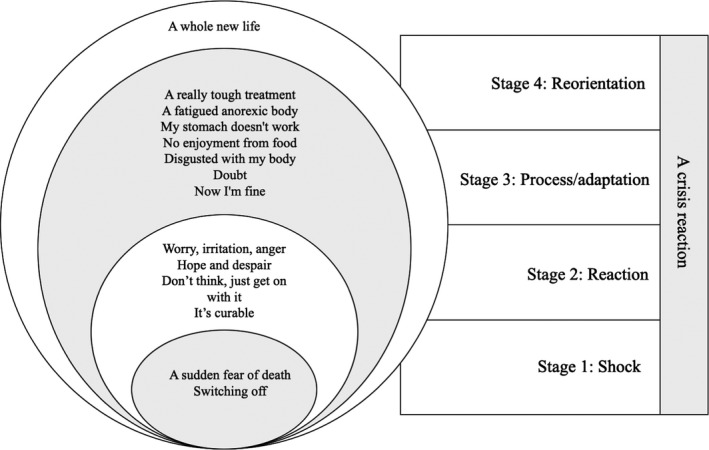
A schematic overview of the 14 sub‐categories derived from the participants' narratives, 6 months following surgery for oesophageal cancer, presented in line with the four stages of a crisis reaction

The results are presented according to the overall theme *Experiencing a crisis reaction* that emerged as the latent content. Three categories (*From emotionally numb to feeling quite alright, From a focus on cure to reflections about a whole new life* and *From a severe treatment to suffering an emaciated, non‐compliant body)* and 14 sub‐categories, derived from the codes, are described and shown in Table 2. The sub‐categories are illustrated in Figure 1, as interpreted in relation to the four stages of a crisis reaction.

**Table 2:** Sub‐categories, categories and a theme from content analysis of narratives about patients' reflections about adaptation from the time of receiving an oesophageal cancer diagnosis to 6 months after curatively intended treatment

Theme	Experiencing a crisis reaction		
Category	From emotionally numb to feeling quite alright	From a focus on cure to reflections about a whole new life	Suffering from a severe treatment and an emaciated, non‐compliant body
Sub‐category	A sudden fear of death “Switching off” Worry, irritation and anger Now I'm fine	It's curable Don't think, just get on with it Hope and despair Doubt A whole new life	A really tough treatment A fatigued anorexic body My stomach doesn't work No enjoyment from food Disgusted with my body

### 3.1 Experiencing a crisis reaction

The patient narratives were rich with latent meaning; thus, data were abstracted to a higher level, from category to theme level, expressing the underlying meaning, that is, the latent content. Throughout the patient narratives, a clear transition in mental focus was described. From the time of diagnosis where patients could focus only on the curable status of the disease and coming treatment, where nothing else mattered, to a more reflective approach where they described the complexity of the new life situation including a reorientation to a whole new life. This theme was interpreted as reflecting that patients were *Experiencing a crisis reaction* (Figure 1), represented in four stages; shock, reaction, process/adaptation and reorientation (Cullberg, 1980). This theme reflects a transition in how participants retrospectively experienced their adaptation with regard to how they had been feeling, suffering and reflecting during the period from diagnosis to 6 months following surgery.

#### 3.1.1 From emotionally numb to feeling quite alright

Findings related to the category *From feeling emotionally numb to feeling quite alright* highlight an emotionally laden adaptation process from remembering feeling emotionally numb close to the time of the diagnosis and operation to later on, at 6 months after treatment, feeling quite alright. Being diagnosed with oesophageal cancer was described as a sudden fear of death, which included an overall feeling of fear and death agony. All thoughts about the future disappeared and the weeks before and after diagnosis were homogeneously described as very psychologically challenging:

Awful, very uncomfortable…when I got the cancer diagnosis…all dreams, all thoughts, everything I had planned to do next week, it totally disappeared. Everything about the future disappeared, it went completely blank. (#16, Man, 68 years)

It took almost three weeks before I found out, that the cancer hadn't spread. Those three weeks, almost three full weeks, those were the worst of my life. It was pretty much a near death experience, you could say. (#04, Man, 65 years)

To cope with the life‐threatening situation, participants described themselves as “switching off” and expressed feelings of emotional numbness, for example, isolation, emptiness, quietness, introversion, loneliness and also an “out of body experience”:

At the beginning I experienced all this, sort of like I was observing myself from outside, this thing about me having cancer and all. (#17, Man, 80 years)

I felt very empty. I didn't really know what to do. I was alone. (#12, Man, 48 years)

Most described feeling worried, ranging from worrying about cancer recurrence to worrying about persistent symptoms, side effects, increasing pain and weight loss. A change in temper was described, where participants experienced that they lost their temper more often now compared with before the cancer diagnosis. Feeling a general irritation that could not fully be explained was described. Feelings of anger were particularly related to bodily changes, for example, feeling thin and weak, side effects of treatment and, anger related to the disease:

Let's just say, you're pretty irritated about looking so bloody thin and disgusting. (#12, Man, 48 years)

Mostly I'm irritated I'm so sodding weak. (#08, Man, 73 years)

At 6 months following the end of treatment, participants described that they were now able to allow previously numbed feelings to come through, describing a sense of feeling fine or quite alright now:

Well, I feel…not like usual, but I feel almost outrageously good, considering what I've been through. (#06, Man, 68 years)

I actually feel very well. (#13, Woman, 52 years)

#### 3.1.2 From a focus on cure to reflections about a whole new life

Findings related to the category *From a focus on cure to reflections about a whole new life* highlight the transition in how the participants reflected back on their experiences during the previous 6 months and how they remembered a limited ability to reflect on their experience soon after the diagnosis but that their reflective capacity improved as time passed. This process described a more cognitive processing of their experiences rather than emotional expression. Participants described that knowing that the tumour was something that could be “removed” from the body increased the focus on “just taking it away” at the beginning of the treatment. Participants described experiencing significant relief when they were informed that the tumour had not spread and that they could undergo curatively intended treatment:

When I was told [there were no metastases], that they couldn't find anything, that was…then I left feeling relieved. (#06, Man 68 years)

Participants described that starting treatment went really quickly and when looking back they reflected that it was satisfying that everything went so fast, because there was no time to think about what they were going through, it just kept moving on. All they could do at this time was focus on the treatment and the operation, everything else was secondary:

Really, this whole period of time has been very intensive. One hasn't really had…had time to think in between, it's been bang, bang, bang. (#19, Woman, 61 years)

Reflecting back on the previous 6 months participants described moving between feelings of hope and despair and that the course of treatment had been challenging and very tough, but in the end worth it:

To see the light, despite it being pitch‐black, you think…don't give up, it's hell for a while, but then it'll be ok. (#12, Man, 48 years)

It was agony. It hurt right, so the only thing you wanted relief from, that was the pain in the end. So, then you think…., well, is it really going to be this hard to have this disease, then I guess…I guess I just have to deal with it. (#05, Man, 74 years)

The participants' descriptions were consistent regarding their reflections about the whole new life situation they were experiencing, requiring excessive readjustments and reorientation. Some participants described difficulties accepting this new way of living and had doubts about having undergone the surgery:

I told them, I will never ever put myself through such a big operation again. Then they laughed, the nurses and said, you won't have to, you already have. There isn't a bigger one. (#13, Woman, 52 years)

However, most described their current situation was a low price to pay for being cured, describing the eye‐opening experience that life goes on and a process of acceptance.

I would do it all again, if it saves my life. Without a doubt. This, these difficulties, they are a small price to pay for living. (#16, Man, 68 years)

It's just this, that I have a bit of trouble accepting…I guess it's the other side of the coin, that's it…Now I just have to accept it's a new way of living, it's a new life. (#04, Man, 65 years)

#### 3.1.3 From a severe treatment to suffering an emaciated, non‐compliant body

Findings related to the category *From a severe treatment to suffering an emaciated, non‐compliant body*, highlight the transition from suffering from undergoing treatment to a focus on suffering the consequences of treatment, that is, the non‐compliant body. Participants described suffering from a tough treatment and the combination of chemotherapy and surgery was described as challenging for both body and mind:

It's more like, hell, it's been tough to go through this [treatment]. (#12, Man, 48 years)

However, participants described that during treatment they could focus only on the treatment step by step and concentrated only on the curable status of the disease, that is, they were going to survive, nothing else mattered. Consequently, it took quite some time before the participants started to realise that the treatment had affected their physical capabilities and also their bodily perceptions. Participants described suffering from the loss of normal eating function, causing nausea, uncomfortable dumping symptoms, gastro‐oesophageal reflux and significant weight loss. Any interest in eating was lost because the food did not taste of anything or tasted disgusting. Participants described they were only eating because they had to and they had lost their feelings of hunger and satiety and no longer got any enjoyment from food:

And I know I must eat and I have to drink, but sometimes it's like, well, sometimes I don't feel like it at all. (#08, Man, 73 years)

It's not like I'm eating because I look forward to it, it's more like eating because I have no choice. (#17, Man, 80 years)

The suffering from fatigue as caused by the cancer and the treatment was described. The fatigue affected the participants both physically and mentally and most expressed the severe suffering from a slowly emaciating body, as a consequence of the disease and treatment:

There's something about the tiredness you can't explain, because it's like……both psychological and physical in some way. (#19, Woman, 61 years)

I get anxiety every time I look in the mirror, when I see my ribs and all that. (#20, Man, 51 years)

The reorientation to the new body also included suffering from new bodily perceptions. Most patients described difficulties getting used to the “loose skin” caused by the rapid postoperative weight loss and the loss of muscle mass probably related to malnutrition and immobility following the operation. To be ashamed of this new body and to be wanting the old body back was repeatedly described:

Well…the body…it's a bit weak, much worse now that I'm so thin, the upper body. (#20, Man, 51 years)

Now there's just nothing left. It's terrible. Maybe I'm a bit vain, but I….I'm ashamed of my body today, I am. (#06, Man, 68 years)

A process of adapting to these new bodily perceptions and the negative feelings associated with the physical adaptation were described. This was also described as negatively affecting participants' sex life and their sexual desire:

You could say, the marital duties were put aside. There was simply no desire. But luckily, it passed and has returned to normal. But, probably, I felt that was the most difficult period of time. (#04, Man, 65 years)

## 4 DISCUSSION

### 4.1 Reflecting a crisis reaction

Participants described an adaptation process over the previous 6 months that included emotional adaptation to both physical and psychological challenges. Participants' descriptions reflected a progression from the time of diagnosis of states of shock, fear, emotional numbness, a focus on the possibility of cure and struggling with a tough treatment to more flexible reflections on adjusting to a changed body and a new life situation at 6 months postoperatively. This process of emotional adaptation was interpreted as reflecting a crisis reaction according to the four stages of shock, reaction, process/adaptation and reorientation (Cullberg, 1980). In line with the shock phase, participants described remembering reacting to their diagnosis with significant fear of death resulting in feelings of emotional numbness to cope with what was to come. In the reaction phase, the shock shifts and access to the emotional life returns (Cullberg, 1980). This stage was reflected in participants' descriptions of recurrent worry and feelings of anger, while at the same time trying to focus only ahead on the possibility of cure and increasing reflective thoughts about their experiences moving between feelings of hope and despair. After the reaction stage, the acute crisis is considered over and the real adaptation to the new life situation begins, characterized by an increasing mental processing of what has occurred (Cullberg, 1980). Participants described their struggles with adapting to their changed body and altered emotional, physical and social situation. Although some feelings of doubt emerged, on the whole participants described a process towards acceptance and feeling quite alright, despite their experiences. The final stage according to the four stages of a crisis reaction is the reorientation phase. In this stage, acceptance and orientation towards the future dominate. Moments of emotional pain may still occur, but these are of a more transient nature. This stage is characterized by the process of reconciliation with the new life situation (Cullberg, 1980). On the whole, a clear shift in the participants' perspectives was observed from their recollections of the time of diagnosis, where they were focused on the curable status of the disease and the treatment only, to a wider perspective described at 6 months postoperatively, where most participants had started to reflect on the impact of the disease and treatment on their current life situation. Participants' descriptions included aspects of reorientation with reflections on the price of their experiences and a work in progress towards accepting the whole new life situation.

### 4.2 Impact on emotional adaptation

Previous studies interviewing patients about their post‐treatment experiences 6 months following oesophageal cancer surgery describe that patients are struggling with readjustment to a new identity, affected by the severe disease and treatment and suggest that a shift in expectations related to eating should be addressed during the recovery process (Clarke et al., 2011). In a focus group study addressing the complex physical, social and emotional changes following surgery adjusting to and accepting an altered self was described (McCorry et al., 2009). Similarly, one previous study carried out 12 months following diagnosis describe the struggle of remapping a body that is greatly limited by physiological, psychological and social difficulties caused by the illness, even though the cancer has been “taken away” (Wainwright et al., 2007). Another study carried out interviews with 15 participants at 12 months postoperatively after upper gastrointestinal surgery. Participants described the first year of recovery as being “pale and grey”. Themes identified included, among others, feelings of doubtfulness and disappointment, never feeling quite well and having to adapt to new circumstances, feelings of being changed. The recovery process was interpreted to reflect a movement between darkness and light (Olsson et al., 2010). In line with these studies, the present results highlight participants' suffering from a severe treatment and the difficulties of adjusting to this new body, a body that was described as fatigued, weak and non‐compliant. These findings are also reflected in longer‐term studies showing that 5 years after surgery, patients still report several problems, for example impaired bodily functions, eating and sleep problems, pain and embarrassment by new shortcomings. All these factors together have a great impact on the patients' quality of life and make it difficult for patients to move on (Malmström et al., 2013).

One study highlights the complexity of the word “cure” when talking about recovery after oesophageal cancer. From a medical perspective, an individual is likely assessed as “cancer free” at the time the tumour is removed from the body and further investigations have confirmed the body free from tumour spread, on a cellular level (Carey, Laws, Ferrie, Young, & Allman‐Farinelli, 2013). However, from a non‐medical perspective, the word cure might sound fortunate and be interpreted as regaining ones pre‐cancer abilities. Unfortunately, this is not always the case for oesophageal cancer survivors and as reflected in the present results, participants described the significant consequences of the treatment on the body and emotional life. In fact other studies show, that following oesophageal cancer surgery, patients may not, even years after surgery, regain their eating functions. Rather, some still struggle with symptoms associated with eating on a daily basis and suffer ongoing physical, emotional and social consequences several years postsurgery (Carey et al., 2013).

Previous research suggests that the way patients with cancer conceptualize their survivorship is associated with emotional distress. Patients describing that they were *Never the Same* after the cancer experience, primarily in a negative way, because of near‐constant worry about recurrence and frustration with residual side effects, were found to report the highest cancer‐related worry and depression (Ratcliff et al., 2018). Longitudinal large‐scale studies investigating psychological distress in patients with oesophageal cancer have shown that around 30% of patients report clinical levels of anxiety and depression at 6 month following surgery (Hellstadius et al., 2017). Although the participants in the present study described their current status as overall “fine” at 6 months after surgery, many negative emotions were described when reflecting on the experience of having had oesophageal cancer. On the whole, a severe recovery process significantly affecting emotional adaptation and quality of life has been described in both qualitative and quantitative studies from 6 months up to 10 years following oesophageal cancer surgery (Derogar & Lagergren, 2012; Hellstadius et al., 2017, 2014; Malmström et al., 2013; Martin et al., 2007; Schandl et al., 2016).

### 4.3 Implications

According to the Swedish Oesophageal‐and Gastric National Care Program (Nationellt vårdprogram matstrup‐ och magsäckscancer, 2017), all patients should be informed, prior to treatment, that oesophagectomy is an extensive surgical procedure demanding vast readjustments, particularly related to eating and consequently also to the patient's social life. However, the national programme also suggests that all patients should be offered psychological consultation if needed and continuous information, when wanted. However, no systematization or clarification of the nature or the timing of the support or information is provided. The need for targeted support programmes or single psychological interventions have been addressed in the literature, as well as the need for directed adequate information batteries in order to reduce suffering and improve emotional adaptation after oesophageal cancer (Hellstadius et al., 2016, 2017; Malmström et al., 2013). However, a prerequisite for any psychological intervention to be successful is that patients are able to put their experiences into words. The present findings suggest patients with oesophageal cancer undergoing curatively intended treatment may be suffering from a crisis reaction during the early postoperative stage. An ongoing crisis reaction may affect patients' capacities to receive, interpret and process information, as well as express their need of support. It is possible that an unprocessed crisis reaction might lead to maladaptation and ultimately poor emotional recovery. Thus, patients, carers and healthcare professionals managing patients with oesophageal cancer, should be aware of a potential crisis reaction response, in order for patients to receive and healthcare professionals provide adequate support throughout the illness trajectory.

### 4.4 Methodological considerations

The findings of this study should be interpreted in light of credibility, dependability and transferability to consider the trustworthiness of the study (Guba, 1981). Credibility refers to the overall approach of the study and was increased by a clear rationale and a thorough description of the whole method including the analysis process to enhance the ability to interpret the findings in the actual context where the study was carried out. Aspects related to the credibility of the analysis refers to the selection of meaning units and the extent to which the categories or themes cover the data. Too broad or narrow meaning units imply a risk of losing the meaning of the data. Furthermore, categories or themes may not fully represent the data, leading to findings that exclude relevant parts of the data or are based on irrelevant parts (Graneheim & Lundman, 2004). By including examples of the process of identifying, condensing and coding meaning units, readers may gain an understanding of the analytic process. Through discussions with other members of the research team, the data could be approached with new perspectives (Graneheim & Lundman, 2004; Tong, Sainsbury, & Craig, 2007). Nevertheless, it is possible that the analytic steps led to findings that do not fully represent the experiences of the respondents. Dependability refers to how much the collected data change over time during the analysis process, as well as the modification of the researchers' decisions during the analysis (Graneheim & Lundman, 2004). This was supported by stepwise replication during the analysis process by, in particular, the first and the last author, but also by all authors regarding the creation of sub‐categories and key categories. In addition, interpretation of the material in relation to trustworthiness was also strengthened by the different competencies and medical expertise represented by a multi‐professional team of authors (e.g. nurses, psychotherapists, oesophageal cancer surgeon and psychologist). Moreover, transferability was retained by using a pre‐defined interview guide to maintain a structure during the interviews. Even though the interviews were considered open, a semi‐structured approach might reduce some potential bias from the interviewer. A trained transcriber outside the project was also consulted for the transcription of the interviews to maintain neutrality. It is also suggested that a purposive sample strategy might be used to collect varied experiences of the studied phenomenon and to facilitate transferability of the results to different patient groups or settings (Guba, 1981). Even though a purposeful sampling strategy was used in the present study, it might be difficult to transfer the results to other cancer groups also suffering from major surgery and severe recovery. This may be due to the specific experiences of eating difficulties among patients with oesophageal cancer, which largely affect their adaptation. In addition, how well the participants represent the whole population of patients who undergo surgery for oesophageal cancer must be considered. For example, eight out of 10 participants in the present study were males, which likely had an impact on the content of the material emerging from the interviews. Also, it might reduce the transferability to other cancer groups that are represented by an equal sex distribution. However, the male predominance shown in the present study reflects the high proportion of males being diagnosed with oesophageal cancer, compared with females. It can be also discussed whether a larger sample would have contributed to a wider range of experiences. However, participants' narratives were rich and comprehensive and contained descriptions and meanings that made it possible to understand what had been experienced. The observation during the study was that saturation was reached relatively quickly and thus the interviews did not carry on beyond 10 participants. One possible explanation for this may be that all participants were interviewed at the same time point during recovery. Data saturation deals with the richness of the data referred to as the collection of data until the point of redundancy (Lincoln & Guba, 1985). The literature suggests that there are no clear roles or recommendations for sample size in qualitative research. When judging the meaningfulness of the findings, the richness of the data and the capabilities to capture the phenomenon may be regarded as more important than the actual sample size (Patton, 2002). It is, however, important to highlight that the interviews were carried out 6 months following surgery, and it could therefore have been difficult for participants to remember, in detail, the course of their adaptation period from diagnosis to 6 months later. There is also the risk that the participants' current situation at the time of the interview could have influenced their memories of the earlier period.

## 5 CONCLUSION

Participants described an adaptation process that was emotionally laden from feeling emotionally numb at diagnosis to feeling quite alright at 6 months postoperatively, a more cognitive reflective process transitioning from a focus on cure to increasing reflections about the new life situation and the suffering associated with a severe treatment to difficulties adapting to the altered body. This adaptation process in the face of physical and psychological challenges were interpreted to reflect four stages of a crisis reaction, shock, reaction, process/adaptation and reorientation. Further research is warranted to explore the consequences of such reactions on patients' need for support during this early recovery period.

## CONFLICT OF INTEREST

The authors report no conflicts of interest.

## ETHICAL APPROVAL

The study was approved by the Regional Ethical Review Board in Uppsala, Sweden (DNR 2016/199).
