# Supplementary material for: Reflecting a crisis reaction: Narratives from patients with oesophageal cancer about the first 6 months after diagnosis and surgery
Source: Nurs Open. 2019 Aug 2;6(4):1471–80. doi: 10.1002/nop2.348 (PMC6805708; doi:10.1002/nop2.348)
Supplement: Supplementary file 1 [file NOP2-6-1471-s001.docx]

**INTERVIEW GUIDE**

*Experiences of oesophageal cancer*

**The time of the diagnosis**

1. Can you tell me about when you were diagnosed with oesophageal cancer?
2. Can you describe the feelings associated with the diagnosis?
3. How do you feel now when we talk about it?

**The time of treatment**

1. Can you tell me about your experiences following the operation/treatment?
2. Can you describe the feelings associated with the operation/treatment?
3. How do you feel now when we talk about it?

**The current Situation**

1. Can you tell me about your current situation?
2. Can you tell me about your feelings now?

**The future**

1. Can you tell me about your thoughts and feelings about the future?

***** *All interview questions were followed up by open ended questions such as “can you tell me more”, “could you please be more specific”, “what did you think”, “how did that make you feel” etcetera.*
